# Assessment of caregiver's stress in people affected by dementia

**DOI:** 10.1002/alz70858_102220

**Published:** 2025-12-25

**Authors:** Pietro Gareri, Lucia Pagano, Elisa Lercara, Luciana Barbara Covello, Filomena Rocca, Ilaria Gareri, Carmela Armeli

**Affiliations:** ^1^ Unit of Frailty Care ‐ Center for Cognitive Impairment and Dementia, Catanzaro, Calabria, Italy; ^2^ Department of Frailty ‐ Center for Cognitive Impairment and Dementia, Catanzaro, Calabria, Italy; ^3^ Unit of Frailty Care ‐ Center for Cognitive Disorders and Dementia Catanzaro Lido ‐ ASP Catanzaro, Catanzaro, Calabria, Italy; ^4^ Mesoraca ‐ ASP Kroton, Kroton, Calabria, Italy; ^5^ Unit of Frailty care ‐ Center for Cognitive Impairment and Dementia ‐ Catanzaro Lido, ASP Catanzaro, Catanzaro, Calabria, Italy; ^6^ School of Geriatrics ‐ University Magna Graecia‐Resident in training, Catanzaro, Calabria, Italy; ^7^ Unit of Frailty Care ‐ Center for Cognitive Disorders and Dementia, Catanzaro Lido, ASP Catanzaro, Catanzaro, Calabria, Italy

## Abstract

**Background:**

There is a growing interest in assessing the impact that Alzheimer's disease has on the family and the caregiver's burden. Our study aimed to identify the most common types of burden and the tools currently used to assess the burden of caring for a person with dementia.

**Method:**

Eighty patients with dementia were assessed. The MMSE was used for the cognition score, ADL and IADL for function assessment, the NPI score for behavioral disorders, and the CBI scale for caregiver stress.

**Result:**

Patients’ mean age was 77.4 ± 4.7 years. They had a mean MMSE score of 15.5 ± 3.7, mean ADL was 3.4 ± 2.8, and mean IADL was 3.5 ± 2.1. Behavioral disorders were present in 77.5% of cases, mainly insomnia, psychomotor restlessness, wandering, and aggressiveness. The most exposed caregiver population comprised women;80% of them were housewives, wives, daughters, or daughters‐in‐law. The caregiver's task changed according to the stages of the disease and included emotional support for the most complex activities of daily life and management of behavioral disorders. Among these, severe insomnia increased stress, frustration, anxiety, and depression in the caregiver.

**Conclusion:**

The main complaint of caregivers is the lack of support from the National Health Service. The results of this study suggest that it would be useful to encourage interventions that make daycare and treatment centers more accessible to older people with dementia. The solutions for reducing the caregiver's stress could be enhancing assistance, strengthening home care, territorial social and healthcare networks, and promoting psychological assistance to the caregiver who is at risk of burnout.

